# Hemizygous Deletion on Chromosome 3p26.1 Is Associated with Heavy Smoking among African American Subjects in the COPDGene Study

**DOI:** 10.1371/journal.pone.0164134

**Published:** 2016-10-06

**Authors:** Ferdouse Begum, Ingo Ruczinski, John E. Hokanson, Sharon M. Lutz, Margaret M. Parker, Michael H. Cho, Jacqueline B. Hetmanski, Robert B. Scharpf, James D. Crapo, Edwin K. Silverman, Terri H. Beaty

**Affiliations:** 1 Department of Epidemiology, Johns Hopkins Bloomberg School of Public Health, Baltimore, Maryland, United States of America; 2 Department of Biostatistics, Johns Hopkins Bloomberg School of Public Health, Baltimore, Maryland, United States of America; 3 Department of Epidemiology, Colorado School of Public Health, Aurora, Colorado, United States of America; 4 Department of Biostatisitics and Informatics, Colorado School of Public Health, Aurora, Colorado, United States of America; 5 Channing Division of Network Medicine and Division of Pulmonary and Critical Care Medicine, Brigham and Women’s Hospital, Harvard Medical School, Boston, Massachusetts, United States of America; 6 Department of Oncology, Johns Hopkins University School of Medicine, Baltimore, Maryland, United States of America; 7 Department of Medicine, National Jewish Health, Denver, Colorado, United States of America; Yale University, UNITED STATES

## Abstract

Many well-powered genome-wide association studies have identified genetic determinants of self-reported smoking behaviors and measures of nicotine dependence, but most have not considered the role of structural variants, such as copy number variation (CNVs), influencing these phenotypes. Here, we included 2,889 African American and 6,187 non-Hispanic White subjects from the COPDGene cohort (http://www.copdgene.org) to carefully investigate the role of polymorphic CNVs across the genome on various measures of smoking behavior. We identified a CNV component (a hemizygous deletion) on chromosome 3p26.1 associated with two quantitative phenotypes related to smoking behavior among African Americans. This polymorphic hemizygous deletion is significantly associated with pack-years and cigarettes smoked per day among African American subjects in the COPDGene study. We sought evidence of replication in African Americans from the population based Atherosclerosis Risk in Communities (ARIC) study. While we observed similar CNV counts, the extent of exposure to cigarette smoking among ARIC subjects was quite different and the smaller sample size of heavy smokers in ARIC severely limited statistical power, so we were unable to replicate our findings from the COPDGene cohort. But meta-analyses of COPDGene and ARIC study subjects strengthened our association signal. However, a few linkage studies have reported suggestive linkage to the 3p26.1 region, and a few genome-wide association studies (GWAS) have reported markers in the gene (*GRM7*) nearest to this 3p26.1 area of polymorphic deletions are associated with measures of nicotine dependence among subjects of European ancestry.

## Introduction

Cigarette smoking is the leading environmental risk factor for many chronic diseases including chronic obstructive pulmonary disease (COPD), coronary artery disease and several common cancers [1, 2]. Different quantities and patterns of exposure to cigarette smoking can reflect different levels of nicotine dependence, in addition to the ease of access to cigarettes and other social forces. Smoking behavior is a complex phenotype because it has both genetic [3, 4] and non-genetic components (including social and environmental factors). To undertand the genetic underpinning of this complex phenotype, several large-scale GWAS have been conducted on quantitative measures of smoking behavior, but the majority of studies included subjects of European ancestry, although there have been a few studies of African-American and Asian populations. These previous studies have identified multiple genetic loci significantly associated with smoking behaviors [4–9].

While many genome-wide and candidate gene studies have identified common and rare single nucleotide polymorphism (SNP) markers associated with quantitative measures of smoking behaviors [10–12], to our knowledge none have focused on the association between copy number variants (CNVs) and quantitative measures of smoking behaviors or nicotine dependence. This study focuses on testing for association between polymorphic CNVs and different phenotypes reflecting smoking behavior in a sample of adult smokers drawn from the COPDGene study, a large study of current and former smokers including both non-Hispanic White (NHW) and African American (AA) adults (aged 45 or over) with at least 10 pack-years exposure to cigarette smoking. Structural variants (including CNVs such as deletions and amplifications of segments of DNA) can be estimated from the genome-wide SNP marker panels used for GWAS, but individuals can have a wide variety of CNVs, differing in both size and location. Polymorphic CNVs, which are deletions/amplifications that are common in a population (above 1% frequency), can be used in tests of association, just like SNPs. We hypothesized that CNVs could be significantly associated with smoking-related phenotypes. However, because CNVs are based on estimated deletions/amplifications, inferences based on significant association tests can be more tenuous, although they can still identify genes potentially influencing complex phenotypes, such as quantitative measures of smoking behavior.

## Materials and Methods

### Ethics Statement

Written informed consent was obtained from all COPDGene study participants, with IRB approvals at the 21 participating clinical centers. The full names of the ethics committee/institutional review boards with the protocol numbers that approved this study are presented in S1 Table.

### Subjects

The COPDGene study (NCT00608764, www.copdgene.org) recruited a total of 10,192 adult smokers [13] and approximately one-third of them are self-identified AA and two-thirds are NHW. This cohort of current and former smokers included individuals ranging across all four severity classes of COPD as defined by the Global Initiative for Chronic Obstructive Lung Disease (GOLD COPD) guidelines[14, 15], as well as individuals with normal pulmonary function and individuals with abnormally restricted spirometric values[16]. COPDGene collected information on detailed personal smoking history for all study subjects, along with other demographic and behavioral risk factors. The complete questionnaire used is available on the COPDGene website [13].

### Phenotypes of Interest

In this study, we considered two quantitative smoking phenotypes: average number of cigarettes smoked per day and pack-years of smoking history. Pack-years is defined as the reported average number of packs of cigarettes smoked per day times the years of smoking. Both of these phenotypes are continuous in nature, but not normally distributed. Therefore, we log-transformed both phenotypes before analysis, in addition to considering categories of the average number of cigarette smoked per day as an ordinal phenotype. We recoded the average number of cigarettes smoked per day phenotype as 1 if 10 or less cigarettes were smoked, 2 if greater than 10 and less or equal to 20 cigarettes were smoked, etc., finally coding category 7 if more than 60 cigarettes per day were smoked.

### Genotyping and Calling Copy Numbers

DNA was extracted from whole blood and genotyped on the Illumina Omni-Express chip. Previous articles on COPDGene genome-wide association analyses reported details of initial quality control (QC) of genotype data [13, 17–23]. GWAS QC steps also included principal components calculation for both AA and NHW groups separately to summarize and adjust for their genetic ancestry. The hidden Markov model (HMM) based PennCNV algorithm was used to estimate CNVs exploiting the log R ratios (LRRs) and B allele frequencies (BAFs) from Illumina’s Omni-Express probes [24]. After CNV calling and QC, there were 9,076 samples (6,187 NHWs; 2,889 AAs) available for analysis of polymorphic CNVs (S1 Fig). Polymorphic CNVs were identified within each racial group, and those CNVs with >1% frequency among all subjects passing QC within each racial group became the genetic markers of interest for this analysis. Details of the QC process and CNV calling steps are discussed in Begum et al. (2015) [25].

### Testing for Association

The genome-wide CNV distribution is very different between AA and NHW subjects, so we estimated all CNV components separately for the two racial groups, and we performed stratified genome-wide CNV association analyses for each group. Our main focus in this analysis was to test for association between polymorphic CNVs and quantitative phenotypes describing smoking behaviors. Calling of CNV components is described in Younkin et al. and Begum et al. [25, 26]. Log_10_-transformed values of pack-years were used for the heavily (right) skewed data (S2 Fig), and we categorized average number of cigarette smoked per day into 7 categories, and considered that as a continuous phenotype. Linear regression models were used to assess the relationship between a quantitative smoking phenotype and polymorphic CNVs while adjusting for covariates such as gender and age. The average admixture score [27], which summarizes the average European ancestry among AA subjects, was also considered in the analysis of all AA subjects.

We then adjusted for multiple comparisons for the number of polymorphic CNVs within each racial group by designing a permutation test. In this permutation, we randomly assigned deletions and their accompanied average admixture score to all AA subjects and computed the minimum p-value for each replicate set. We repeated this procedure 10,000 times to establish the genome-wide significance level with a family-wise error rate of 5%; and the nominal threshold was set at 3.50, which is the 5th percentile -log_10_(p) maxima over all 10,000 replicates. A test result was considered significant if it was lower than this threshold. Besides the PennCNV analysis, we used R to carry out our association analyses for this study (http://cran.r-project.org/).

## Results

Out of 10,192 COPDGene study subjects, 9,970 passed initial GWAS QC. Additional QC for CNV estimation excluded 894 subjects, leaving 9,076 subjects (2,889 AAs; 6,187 NHWs) for analysis of polymorphic CNVs. Table 1 presents the demographic characteristics and distribution of the smoking phenotypes among males and females for these 2,889 AA and 6,187 NHW subjects. None of our genome-wide CNV association results was significant in the larger group of 6,187 NHW COPDGene subjects, so we here limit our subsequent discussion to AA subjects only.

**Table 1:** Demographic characteristics and smoking behavior distributions among African-American and Non Hispanic White study subjects from COPDGene.

	African-American (AA) Total (Male: Female)	Non Hispanic White (NHW) Total (Male: Female)
Sample size	2889 (1648 M: 1241 F)	6187(3262 M: 2925 F)
Mean age at enrollment	54.5(54.3 M: 54.8 F)	62.1 (62.4 M: 61.7 F)
Percent current smokers (%)	80.5(82.9 M: 77.2 F)	39.3 (40.25 M: 38.22 F)
Mean pack-years of smoking	38.1 (38.8 M: 37.2 F)	47.3 (51.6 M: 42.55 F)
Average cigarette smoked per day	21.2 (21.5M: 20.8F)	25.8(27.6 M: 23.8 M)

### Polymorphic CNV Association Analysis of Pack-years of Smoking in AA Subjects

**Fig 1:**
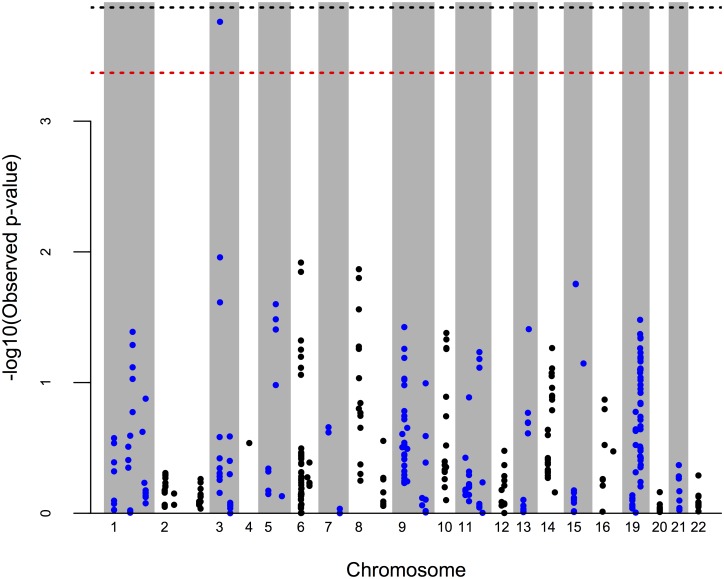
Genome-wide CNV association scan for pack-years in African American COPDGene subjects: Each point represents a CNV component estimated by PennCNV. The red dotted line at 3.5 represents the genome-wide significance levels needed to maintain a family-wise error rate (FWER) of 5%, obtained via 10,000 permutation tests. The black dotted line at 3.87 represents the genome-wide significance levels for Bonferroni correction. 22 vertical bands indicate the 22 autosomes examined.

**Fig 2:**
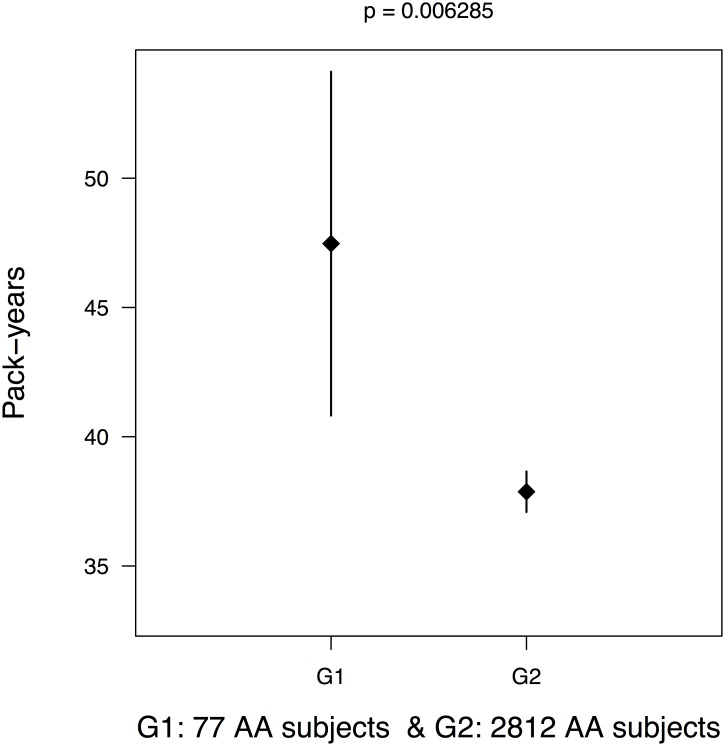
Mean and 95% Confidence Interval (CI) plot of pack-years for two groups of AA subjects. a. Mean pack-years and 95%CI in 77 hemizygous deletion carriers vs. rest of the subjects(2812) in chr 3p26.1. The observed mean difference in pack-years was statistically significantly (p = 0.00629) between these two groups.

Considering only polymorphic CNV components (i.e. those with >1% frequency among AA subjects), there were genome-wide 373 CNV components encompassing 22 autosomes available for association testing among all 2,889 AA subjects. We adjusted for age, gender and admixture scores (i.e., the estimated average European ancestry among AA subjects) in testing for association with pack-years of smoking. Fig 1 shows the Manhattan plot of 373 polymorphic CNV components, and its corresponding QQ plot is presented in S3 Fig. After multiple comparison adjustment, only one polymorphic CNV component on chromosome 3p26.1 (spanning 1,691 bp from position 6212573 to 6214264, hg18) showed genome-wide significance (*p* = 0.00017; -log_10_(0.00017)~3.77) (S4 Fig). The raw intensity data (LRRs and BAFs) at chr3p26.1 were of high quality, and all CNV calls were made with high confidence (see S5 Fig). Fig 2 shows the mean pack-years and 95% confidence interval (CI) plots for different polymorphic CNV counts between 77 hemizygous deletion carriers and the remaining AA subjects for this region of chr 3p26.1. Mean pack-years was statistically significantly different (*p* = 0.00629) between 77 hemizygous deletion carriers and rest of the AA individuals.

Although one polymorphic CNV component of size 1,691bp, (occuring as a hemizygous deletion in 77 AA subjects) was significant in our permutation analysis, further investigation showed this 1,691bp deletion is part of a larger section of polymorphic deletions on chr3p26.1 spanning approximately 20kb (6214264–6194430 = 19,834bp). There were 40 additional AA subjects carrying smaller hemizygous deletions upstream of this region, bringing the total number of hemizygous deletion carriers to 117. Among all 117 subjects with any estimated deletion in this region, the 77 subjects carrying larger deleted segments of 1,691bp showed significant association. This region is an intergenic region, which includes some microRNAs, and the nearest known gene is *GRM7* (chr3: 6,877,927–7,758,217; Size: 880kb) which is approximately 600kb away from the most significant polymorphic CNV segment observed here.

The demographic and clinical characteristics of 2,772 normal diploid subjects and 117 subjects with hemizygous deletions (77 of those 117 with larger hemizygous deletions, spanning the significant polymorphic CNV component and 40 other individuals with shorter deletions) are presented in Table 2. We did not observe any significant difference between these two groups of hemizygous deletion carriers for demographic and clinical characteristics. However, the pack-years of smoking was significantly different (*p* = 0.0006055) between the 77 carriers with larger deletions and the 40 AA subjects carrying smaller deletions.

**Table 2:** Details on 2,772 AA normal diploid subjects and 117 AA subjects with hemizygous deletions on chr 3p26.1 (77 of these 117 AA subjects carryied CNV components achieving genome-wide significance had larger hemizygous deletions (spanning an additional 1.6kb), while 40 of 117 AA subjects had shorter hemizygous deletions in this section).

	2772 AA normal diploid subjects	40 subjects with shorter deletions	77 subjects with larger deletions
**Gender**			
** Male**	1567 (56.5%)	24 (60%)	57 (74%)
** Female**	1205 (43.5%)	16 (40%)	20 (26%)
**Current smoker**	**2230 (80.4%)**	**35 (87.5%)**	**60 (77.9%)**
** Male**	1302 (58.4%)	20 (57.1%)	45 (75%)
** Female**	928 (41.6%)	15 (42.9%)	15 (25%)
**Ex-smoker**	**542 (19.6%)**	**5 (12.5%)**	**17 (22.1%)**
** Male**	265 (48.9%)	4 (80%)	12 (70.6%)
** Female**	277 (51.1%)	1 (20%)	5 (29.4%)
**Average smoking per day**	**21.1**	**19.0**	**25.7**
** Male**	21.4	19.5	25.7
** Female**	20.8	18.3	25.9
**Pack-years of smoking**	37.9	33.3	47.5
**Mean age at enrollment**			
** Male**	54.4	52.7	53.2
** Female**	54.9	54.9	52.5
**COPD status**			
** Cases**	2000 (72.1%)	27 (67.5%)	59 (76.6%)
** Controls**	565 (20.4%)	8 (20.0%)	14 (18.2%)
** Unknowns**	207 (7.5%)	5 (12.5%)	4 (5.2%)

We also looked at COPDGene 6,187 NHW subjects and found there were only 3 hemizygous deletion carriers and one subject with an amplification in 3p26.1 (spanning 1,691 bp from position 6212573 to 6214264, hg18), which shows this CNV component is specific to people of AA ancestry.

### Polymorphic CNV Association Analysis of Average Cigarette Smoking Per Day Among AA Subjects

The polymorphic CNV component on chromosome 3p26.1 associated with pack-years of smoking also showed border-line significance (p = 0.000254, -log_10_(p) = 3.59) for the average cigarette smoking per day phenotype among all 2,889 AA subjects (S6 Fig). Again, age and gender, plus average admixture scores (i.e., the estimated percent European ancestry among AA subjects) were included as covariates.

### Replication in Atherosclerosis Risk in Communities (ARIC) Study

To validate our CNV estimations on this region of chromosome 3p26.1 (6194430bp to 6214264bp), we further investigated CNV estimation in the ARIC study [28], which included 9,043 European American (EA) subjects and 3,210 African American (AA) subjects drawn from multiple communities. Genotyping for the ARIC study was done on a different platform (the Affymetrix 6.0 chip), but PennCNV was also used to estimate CNVs [29]. Copy number estimates from a mixture model fit to the median LRR from this region among all ARIC AA subjects revealed: 3 homozygous deletions, 170 hemizygous deletions, and 3,037 normal diploid genotypes. Similarly, the CNV component counts for the specific 1,691 bp deletion achieving genome-wide significance in the COPDGene study identified 3 homozygous deletion carriers and 145 hemizygous deletion carriers among all ARIC AA subjects. Therefore, the counts of CNVs from the ARIC study among AAs are quite comparable with those seen in COPDGene, confirming this polymorphic deletion is more common among AA subjects. Among AA subjects in the ARIC cohort (a less selected cohort of adults, including many non-smokers), however, only 52.6% were ever smokers, whereas COPDGene study subjects were all smokers with at least 10 pack-years exposure. After sub-setting the ARIC cohort into those subjects with at least 10 pack-years of smoking, only 973 AA subjects were left for analysis, but the mean pack-years of smoking 29.8 was lower in the ARIC study compared to mean pack-years of smoking 38.1 for COPDGene study subjects. Among these 973 AA subjects, 47 were hemizygous deletion carriers and the remaining were normal diploids. Similar regression analysis of pack-years of smoking on polymorphic CNVs in this sub-set of AA subjects from ARIC showed no significant association between this CNP component on 3p26.1 and pack-years of smoking (p = 0.115), although the direction of the estimated effect size was the same (see S2 Table).

While we were not able to replicate this result in the ARIC study, a SNP (rs6762258) on chromosome 3 in *GRM7* was marginally associated with the log_10_(pack-years) adjusting for age, gender, and genetic ancestry via PCAs (p = 0.004) among AA in the COPDGene study. This SNP (rs6762258) showed almost no correlation with the CNV component of interest (correlation coefficient = 0.04; p = 0.03).

We also performed meta-analysis of ARIC and COPDGene AA study subjects for the pack-years of smoking phenotype which improved the significance (*p* = 6.126e-05; -log_10_(6.126e-05)~4.21) compared to the p-values obtained from COPDgene study alone (*p* = 0.00017; -log_10_(0.00017)~3.77). We checked for association between polymorphic CNVs with the average cigarette smoked per day among AAs in the ARIC study. The average cigarette smoked per day among AAs in ARIC was 19.2, and was slightly lower than cigarettes per day reported by AA subjects in COPDGene study 21.2. Although the reported average cigarettes smoked per day was not significantly associated with the polymorphic CNV on chr. 3p26.1 (p = 0.221) in the ARIC study (see S2 Table), a meta-analysis combining AA subjects from ARIC and COPDGene study improved the significance (*p* = 0.0001577; -log_10_(0.0001577)~3.80) compared to the p-values obtained from COPDgene study alone (p = 0.000254, -log_10_(p) = 3.59).

## Discussion

In this study, we performed a genome-wide association study of polymorphic copy number variants (CNVs) and quantitative measures of smoking behavior using subjects from the COPDGene study cohort of 9,076 subjects (stratified by race). We performed stratified analysis in NHW (n = 6,187) and AA (n = 2,889) subjects separately, but we only found genome-wide significant evidence of association between polymorphic CNVs on chromosome 3p26.1 and smoking behavior among African American subjects, where these CNVs are far more polymorphic. Deletions in this 1.6 kb region were significantly associated with two quantitative measures of smoking behavior: pack-years of smoking and average number of cigarette smoked per day. Hemizygous deletion carriers were more common among AA subjects (a total of 117 but 77 had larger deletions driving the association signal) compared to NHW subjects, i.e. there were only 3 NHW subjects with a hemizygous deletion and one subject with one copy amplification in that area among a total of 6,187 NHW subjects in COPDGene study and 7 EA subjects with hemizygous deletion and 21 subjects with single copy gain in the ARIC study. People of African ancestry often have more polymorphic CNVs and other forms of genetic markers compared to people of European ancestry [30, 31]. African Americans in the ARIC[32] study showed similar counts of hemizygous deletion carriers and normal diploids, but the substantial differences in the quantity of exposure to cigarette smoking in the community-based ARIC cohort severely limited statistical power to replicate this association seen in COPDGene. When we used a similar regression model on heavy smokers among African American ARIC participants, there were fewer than 1,000 subjects with available phenotype and CNV data, so it is not surprising that no significant results were obtained. A formal power analysis supported this claim (see S8 Fig). Nonetheless, this finding of association between polymorphic deletions on chromosome 3p26.1 may help to better understand genetic factors controlling smoking behavior.

There are several genome-wide studies of SNP markers where smoking behaviors (including pack-years, nicotine dependence, etc.) have been used as outcome phenotypes. Several have achieved genome-wide significance among European Americans, but fewer among African Americans. In a recent meta-analysis of four smoking behavior phenotypes, David et al. [33] identified one SNP in chr 15q25.1 achieving genome-wide significance in a combined sample of 15,547 African American subjects from 13 studies with the phenotype cigarettes per day. This region of chr15q25.1 has shown strong and consistent evidence of association with nicotine dependence, smoking intensity and lung cancer risk in samples of European ancestry. These authors suggest this one SNP achieving genome-wide significance may define a single common haplotype exerting some effect on smoking behaviors. Among the second-tier hits identified in this meta-analysis were some novel genes, but none could account for much of the variance in quantitative measures of smoking behavior. In a candidate gene approach, Saccone et al. [34] confirmed a well-recognized association between a non-synonymous, functional SNP (rs16969968) in chr 15q25 and nicotine dependence in both European Americans and African Americans in a case-control study of confirmed smokers, but other markers in this region showed more complex statistical evidence of association, as well as greater heterogeneity between racial groups. Gelernter et al. [5] looked at nicotine dependence in a GWAS using 2,114 European American and 2,602 African American smokers, although most genome-wide significant SNPs were only supported in one racial group. In part, this difference in evidence of association between smoking behavior phenotypes in GWAS or candidate gene studies may reflect background differences in linkage disequilibrium (LD) patterns between people of European and African ancestry, but also differences in marker allele frequencies and differences in sample sizes become important (where studies of African Americans are typically smaller). Hamidovic et al. [35] showed a quantitative assessment of smoking persistence (based on pack-years) also revealed different patterns of association with SNPs between European and African ancestry subjects from the ARIC study cohort. However, in an analysis of the MESA Lung cohort, Powell et al. [36] could not demonstrate any differences across four racial/ethnic groups in the association between smoking behaviors and quantitative measures of spirometric lung function or emphysema.

There are no published studies of polymorphic CNVs influencing quantitative measures of smoking behaviors directly in either racial group, but linkage analysis studies from “The Nicotine Addiction Genetics linkage project” showed this chromosome 3p26-25 region was linked to depression with a multiponint LOD score of 4.14 in heavy smoker families, highlighting the need for further investigation into this genomic region [37, 38]. Here, we explored the effects of common deletions/amplifications on pack-years and reported number of cigarettes per day. Only the smaller African American sub-group achieved genome-wide significance, perhaps because they have more hemizygous deletion carriers (117) on chr3p26.1 compared to NHW (where there was only 3 hemizygous deletion carriers and one duplication carrier). The nearest gene to our most strongly associated CNV component on chromosome 3p26.1 deletion region is *GRM7*, which is involved with glutamate receptor activation. Several previous GWAS studies of nicotine dependence-related phenotypes have reported associations to markers in *GRM7* [39–42]. Examining gene expression levels of *GRM7* in the GTEx portal (http://www.gtexportal.org/home/gene/GRM7) showed higher expression in different parts of brain including the cortex, hypothalamus and hippocampus (S7 Fig), which included 84.3% NHW and 13.7% African Americans in their expression analysis. Liu et al.[40] performed a combined analysis of pooled DNA samples from both European and African American subjects in a case-control study of substance dependence, and *GRM7* was among the many genes showing some evidence of allele frequency differences between cases and controls. A related gene (*GRM8* on chr. 7) was identified in another study of heroin addiction, but the relationship between quantitative measures of smoking behavior and other substance abuse remains tenuous [43]. This is the first study of polymorphic structural variants (CNVs) and quantitative measures of smoking behavior, and the first time any association between polymorphic CNVs on chromosome 3p26.1 has been reported to be associated with relatively heavy smokers among African American subjects.

## Supporting Information

S1 Fig Data QC pipeline: 10,192 subjects were genotyped with the Illumina OmniExpress chip. After QC 9,970 subjects were included in GWAS analysis. Among those subjects, 9,149 passed PennCNV QC. Due to poor CNV calling, an additional 73 subjects from 4 plates were excluded. 9,076 subjects were included in CNV analysis (2889 AA subjects and 6187 NHW subjects).
(PDF)

S2 Fig Distribution of pack-years and log-transformed pack-years among COPDGene AA subjects. (PDF)

S3 Fig Quantile-Quantile (Q-Q) plot (λ = 1.02) of the genome-wide CNP association study with pack-years of smoking among AA subjects. –log_10_ transformed observed p-values (Y-axis) were plotted against–log_10_ transformed expected p-values (X-axis).
(PDF)

S4 Fig Histogram of 10,000 permuted p-values and the observed p-values (denoted by the red dotted line). (PDF)

S5 Fig B allele frequency (BAF) and Log R Ratio (LRR) plot of deleted and normal subjects. **Top panel: a.** BAF plot of observed markers from 77 subjects with hemizygous deletions **b.** LRR intensities for these same 77 subjects with hemizygous deletions **Bottom panel: a.** BAF plot of 77 individuals called as normal diploids in this same region **b.** LRR plot of 77 diploid subjects.
(PDF)

S6 Fig Association plot and QQ plot (λ = 1.05) or association of copy number variants and average cigarettes smoked per day for COPDGene AA subjects. (PDF)

S7 Fig Gene expression for GRM7. **Data Source: GTEx Analysis Release V6 (dbGaP Accession phs000424.v6.p1)** (http://www.gtexportal.org/home/gene/GRM7).
(PDF)

S8 Fig Power analysis plot: As mentioned in the Discussion section of the manuscript, we feel that differences in the quantity of exposure to cigarette smoking in the community-based ARIC cohort severely limited statistical power to replicate our findings. To support this claim, we have conducted the following power analysis. We generated the outcome (log transformed pack-years of smoking history) from a normal distribution based on the CNV of interest for varying effect sizes (Beta ranging from 0 to 1 by 0.005) for 2,889 subjects, the sample size of COPDGene AA subjects, and 973 subjects, the sample size of the ARIC study for subjects with at least 10 pack-years of smoking history. As seen in this figure, there was substantially more power to detect an association among the sample size of the COPDGene study as compared to the sample size from the AIRC study.
(PDF)

S1 Table Estimated regression coefficients. (PDF)

S2 Table Estimated regression coefficients from linear regression models testing for association between pack-years/cigarettes per day and polymorphic deletion CNVs on chr 3p26 in COPDGene & ARIC African Americans. (PDF)
