# Supplementary material for: Hemizygous Deletion on Chromosome 3p26.1 Is Associated with Heavy Smoking among African American Subjects in the COPDGene Study
Source: PLoS One. 2016 Oct 6;11(10):e0164134. doi: 10.1371/journal.pone.0164134 (PMC5053531; doi:10.1371/journal.pone.0164134)
Supplement: S1 Fig — After QC 9,970 subjects were included in GWAS analysis. Among those subjects, 9,149 passed PennCNV QC. Due to poor CNV calling, an additional 73 subjects from 4 plates were excluded. 9,076 subjects were included in CNV analysis (2889 AA subjects and 6187 NHW subjects). (PDF) [file pone.0164134.s001.pdf]

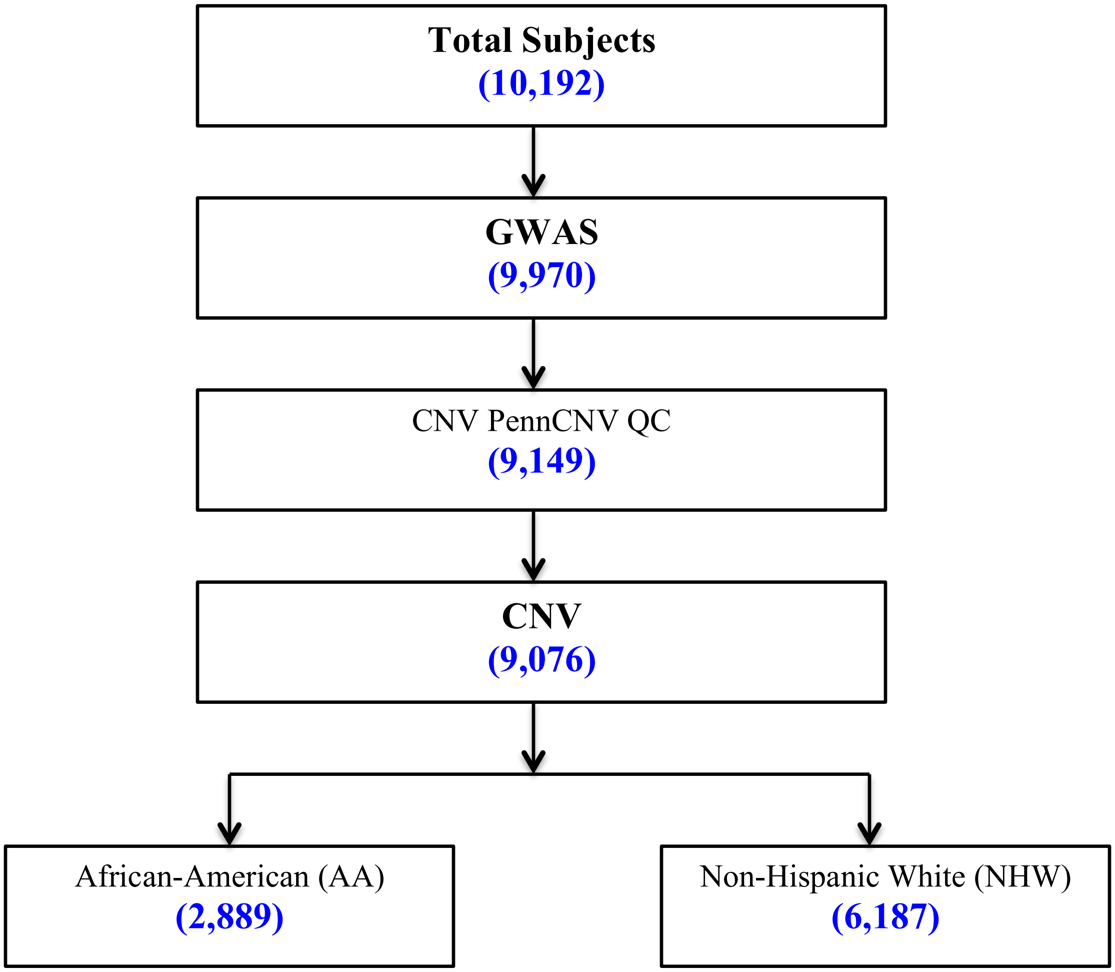

S1 Fig: Data QC pipeline: 10,192 subjects were genotyped with the Illumina OmniExpress chip. After QC 9,970 subjects were included in GWAS analysis. Among those subjects, 9,149 passed PennCNV QC. Due to poor CNV calling, an additional 73 subjects from 4 plates were excluded. 9,076 subjects were included in CNV analysis (2889 AA subjects and 6187 NHW subjects).
