# Supplementary figures and images for: Hemizygous Deletion on Chromosome 3p26.1 Is Associated with Heavy Smoking among African American Subjects in the COPDGene Study
Source: PLoS One. 2016 Oct 6;11(10):e0164134. doi: 10.1371/journal.pone.0164134 (PMC5053531; doi:10.1371/journal.pone.0164134)

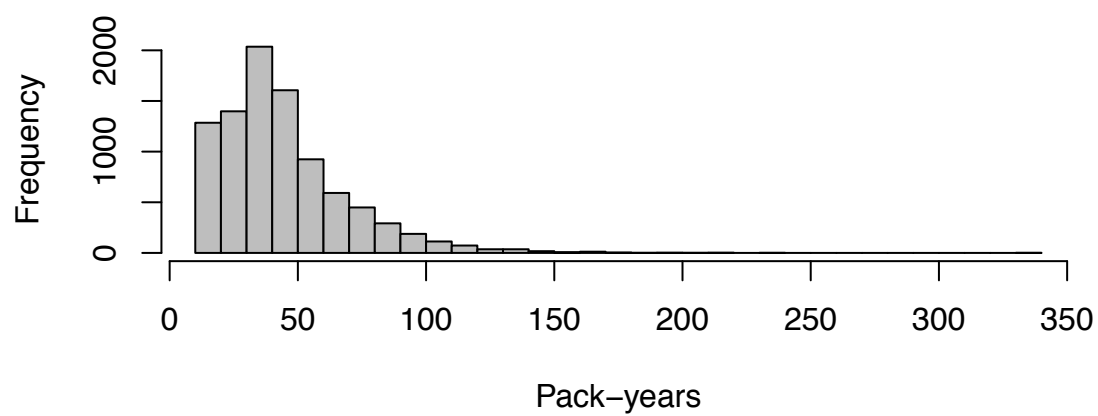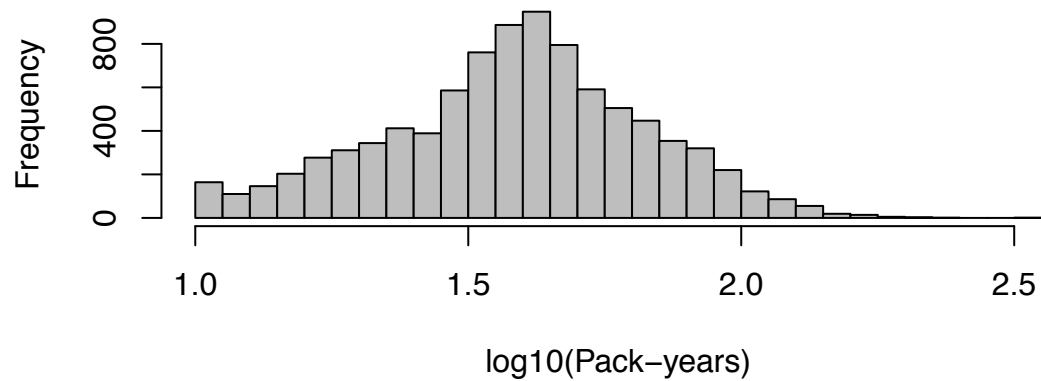

**S2 Fig: Distribution of pack-years and log-transformed pack-years among COPDGene AA subjects**

Supplement: S2 Fig — (PDF) [file pone.0164134.s002.pdf]

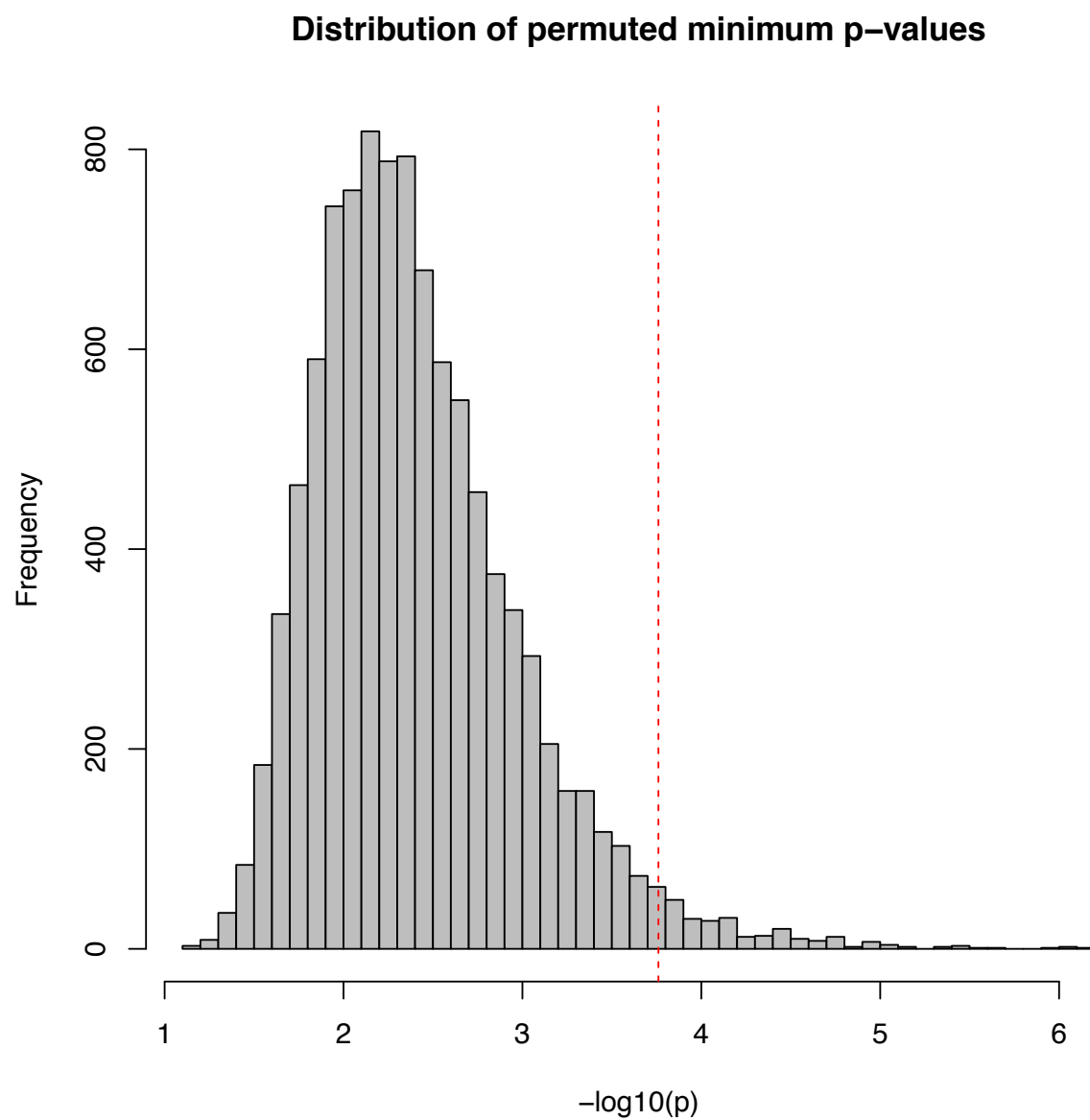

**S4 Fig: Histogram of 10,000 permuted p-values and the observed p-values (denoted by the red dotted line).**

Supplement: S4 Fig — (PDF) [file pone.0164134.s004.pdf]
