# Supplementary material for: Hemizygous Deletion on Chromosome 3p26.1 Is Associated with Heavy Smoking among African American Subjects in the COPDGene Study
Source: PLoS One. 2016 Oct 6;11(10):e0164134. doi: 10.1371/journal.pone.0164134 (PMC5053531; doi:10.1371/journal.pone.0164134)
Supplement: S3 Fig — –log10 transformed observed p-values (Y-axis) were plotted against–log10 transformed expected p-values (X-axis). (PDF) [file pone.0164134.s003.pdf]

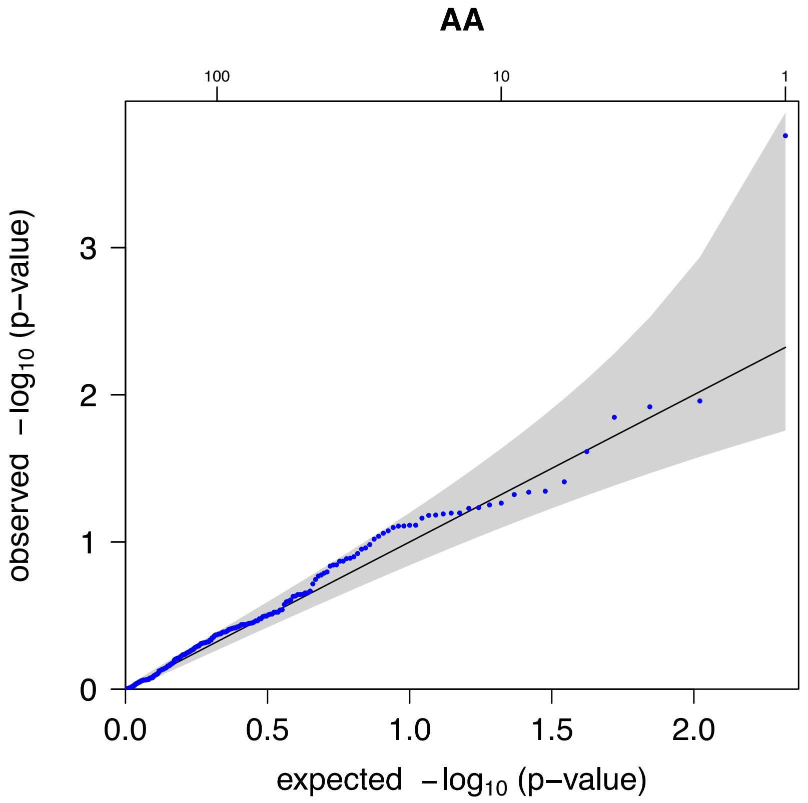

S3 Fig: Quantile-Quantile (Q-Q) plot (λ=1.02) of the genome-wide CNP association study with pack-years of smoking among AA subjects. $-\log_{10}$ transformed observed p-values (Y-axis) were plotted against $-\log_{10}$ transformed expected p-values (X-axis).
