# Supplementary material for: Hemizygous Deletion on Chromosome 3p26.1 Is Associated with Heavy Smoking among African American Subjects in the COPDGene Study
Source: PLoS One. 2016 Oct 6;11(10):e0164134. doi: 10.1371/journal.pone.0164134 (PMC5053531; doi:10.1371/journal.pone.0164134)
Supplement: S5 Fig — Top panel: a. BAF plot of observed markers from 77 subjects with hemizygous deletions b. LRR intensities for these same 77 subjects with hemizygous deletions Bottom panel: a. BAF plot of 77 individuals called as normal diploids in this same region b. LRR plot of 77 diploid subjects. (PDF) [file pone.0164134.s005.pdf]

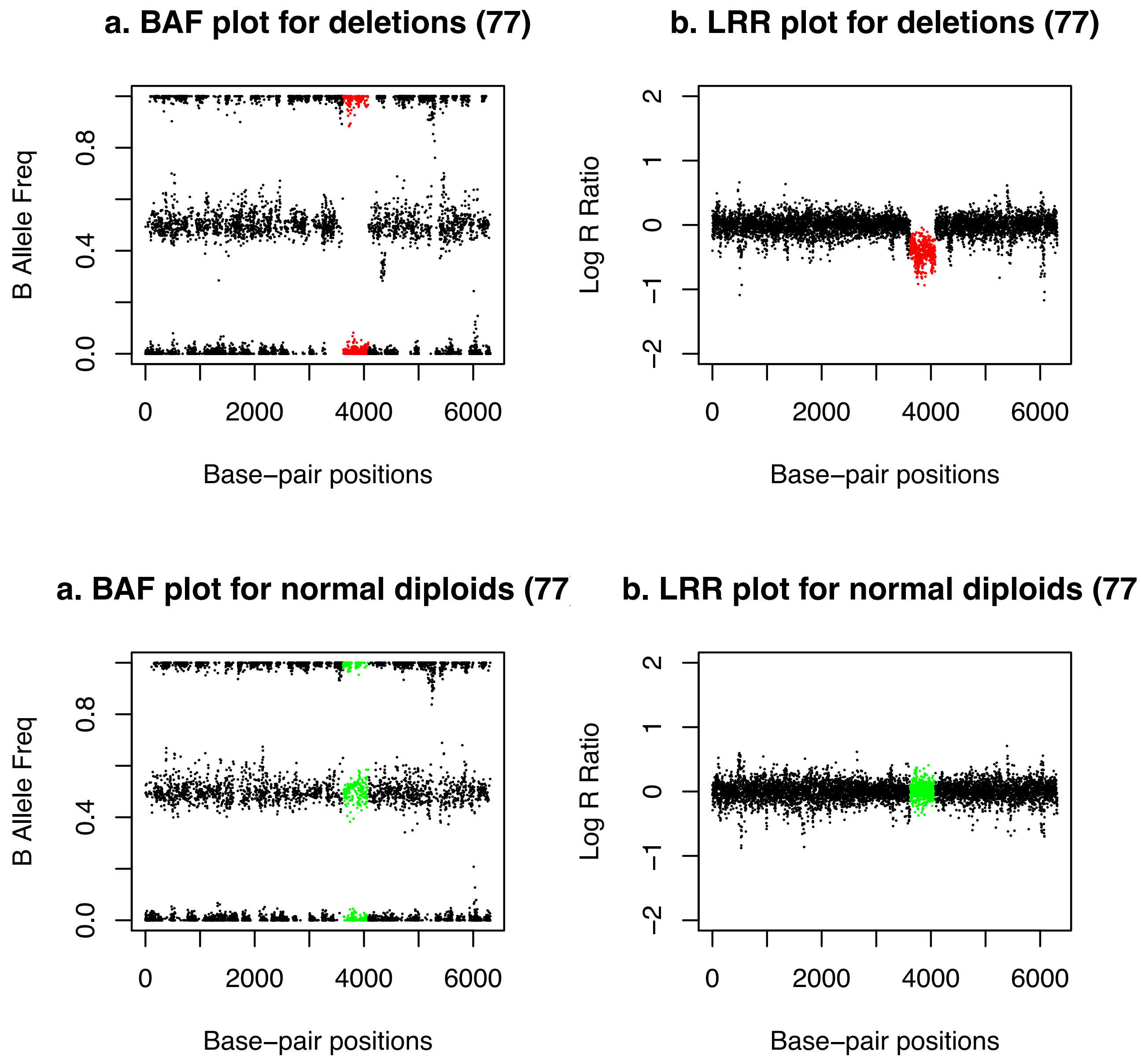

S5 Fig: B allele frequency (BAF) and Log R Ratio (LRR) plot of deleted and normal subjects. **Top panel: a.** BAF plot of observed markers from 77 subjects with hemizygous deletions **b.** LRR intensities for these same 77 subjects with hemizygous deletions
Bottom panel: a. BAF plot of 77 individuals called as normal diploids in this same region **b.** LRR plot of 77 diploid subjects.
