# Supplementary material for: Hemizygous Deletion on Chromosome 3p26.1 Is Associated with Heavy Smoking among African American Subjects in the COPDGene Study
Source: PLoS One. 2016 Oct 6;11(10):e0164134. doi: 10.1371/journal.pone.0164134 (PMC5053531; doi:10.1371/journal.pone.0164134)
Supplement: S6 Fig — (PDF) [file pone.0164134.s006.pdf]

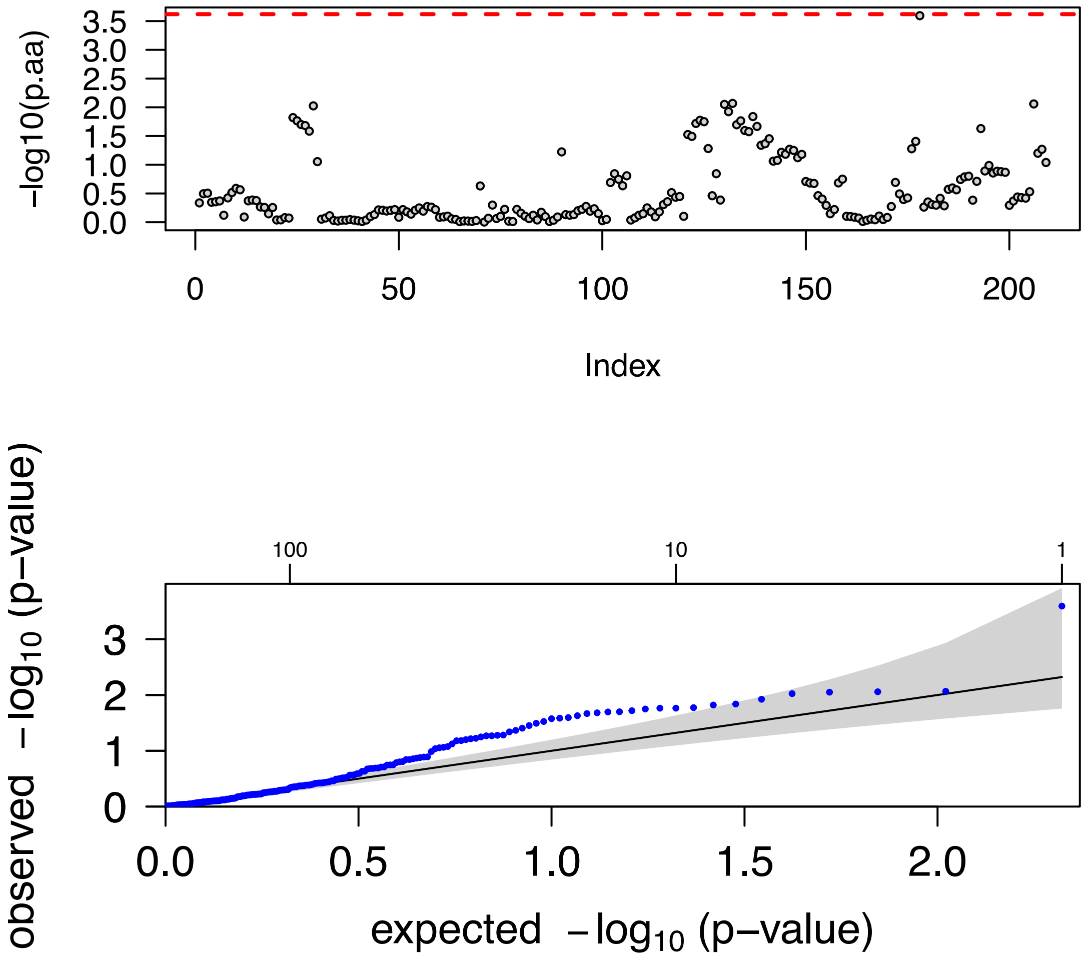

S6 Fig: Association plot and QQ plot (λ=1.05) or association of copy number variants and average cigarettes smoked per day for COPDGene AA subjects.
