# Supplementary material for: Hemizygous Deletion on Chromosome 3p26.1 Is Associated with Heavy Smoking among African American Subjects in the COPDGene Study
Source: PLoS One. 2016 Oct 6;11(10):e0164134. doi: 10.1371/journal.pone.0164134 (PMC5053531; doi:10.1371/journal.pone.0164134)
Supplement: S7 Fig — Data Source: GTEx Analysis Release V6 (dbGaP Accession phs000424.v6.p1) (http://www.gtexportal.org/home/gene/GRM7). (PDF) [file pone.0164134.s007.pdf]

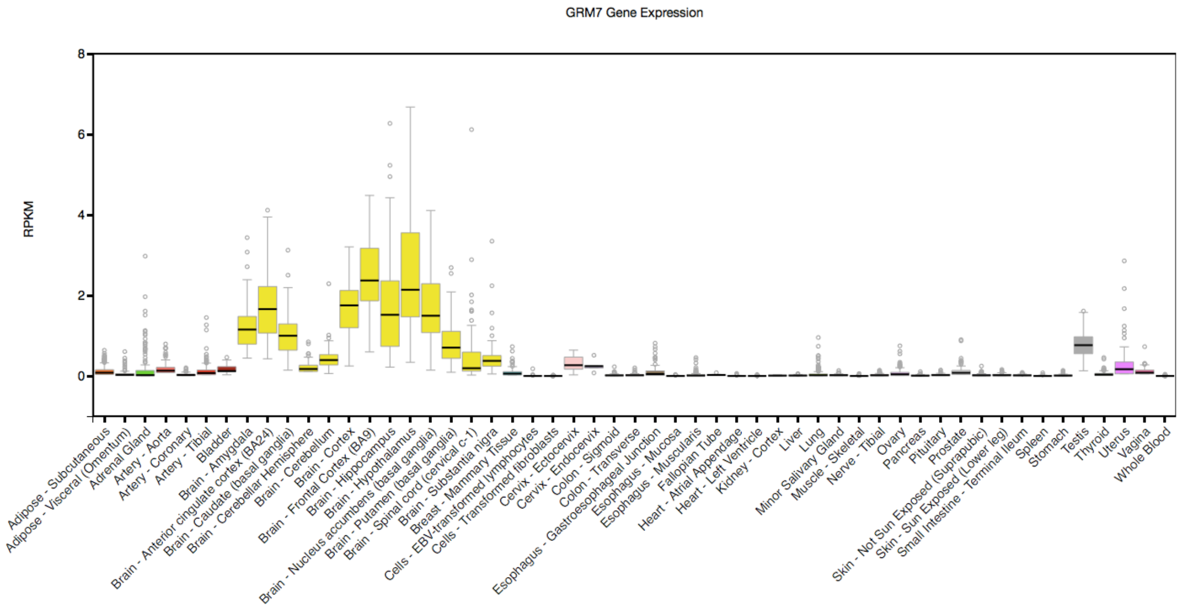

S7 Fig: Gene expression for *GRM7*. Data Source: GTEx Analysis Release V6 (dbGaP Accession phs000424.v6.p1) (http://www.gtexportal.org/home/gene/GRM7)
