# Supplementary material for: Hemizygous Deletion on Chromosome 3p26.1 Is Associated with Heavy Smoking among African American Subjects in the COPDGene Study
Source: PLoS One. 2016 Oct 6;11(10):e0164134. doi: 10.1371/journal.pone.0164134 (PMC5053531; doi:10.1371/journal.pone.0164134)
Supplement: S8 Fig — To support this claim, we have conducted the following power analysis. We generated the outcome (log transformed pack-years of smoking history) from a normal distribution based on the CNV of interest for varying effect sizes (Beta ranging from 0 to 1 by 0.005) for 2,889 subjects, the sample size of COPDGene AA subjects, and 973 subjects, the sample size of the ARIC study for subjects with at least 10 pack-years of smoking history. As seen in this figure, there was substantially more power to detect an association among the sample size of the COPDGene study as compared to the sample size from the AIRC study. (PDF) [file pone.0164134.s008.pdf]

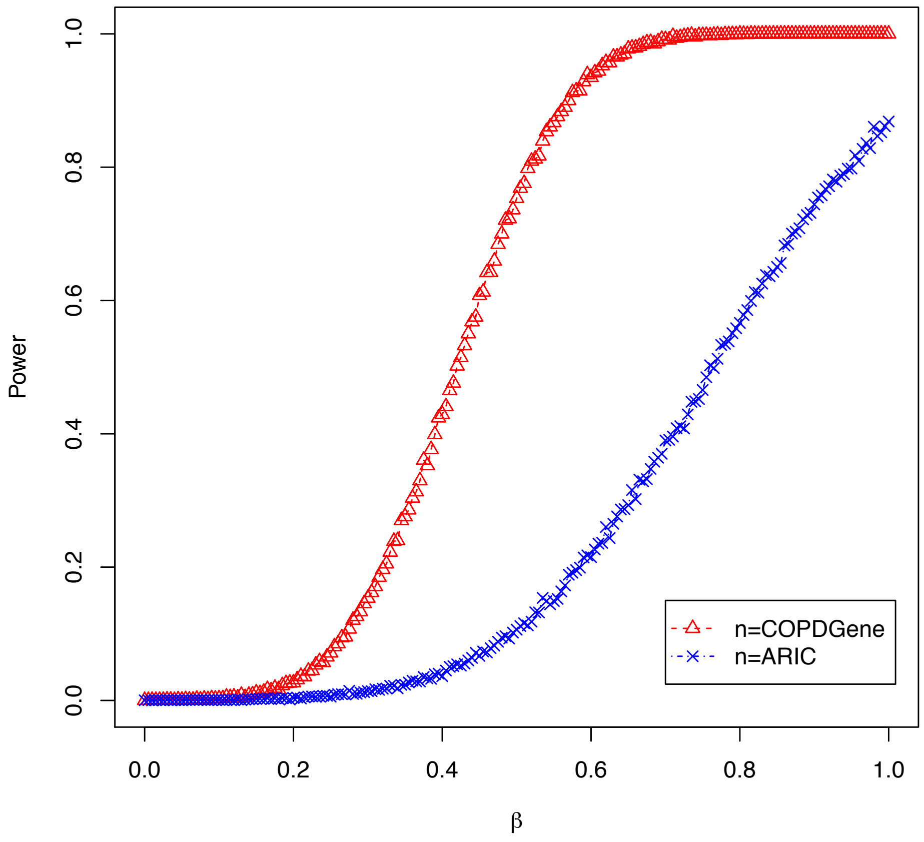

S8 Fig: Power analysis plot. As mentioned in the Discussion section of the manuscript, we feel that differences in the quantity of exposure to cigarette smoking in the community-based ARIC cohort severely limited statistical power to replicate our findings. To support this claim, we have conducted the following power analysis. We generated the outcome (log transformed pack-years of smoking history) from a normal distribution based on the CNV of interest for varying effect sizes (Beta ranging from 0 to 1 by 0.005) for 2,889 subjects, the sample size of COPDGene AA subjects, and 973 subjects, the sample size of the ARIC study for subjects with at least 10 pack-years of smoking history. As seen in this figure, there was substantially more power to detect an association among the sample size of the COPDGene study as compared to the sample size from the AIRC study.
