# Supplementary material for: Hemizygous Deletion on Chromosome 3p26.1 Is Associated with Heavy Smoking among African American Subjects in the COPDGene Study
Source: PLoS One. 2016 Oct 6;11(10):e0164134. doi: 10.1371/journal.pone.0164134 (PMC5053531; doi:10.1371/journal.pone.0164134)
Supplement: S2 Table — (PDF) [file pone.0164134.s010.pdf]

S2 Table: Estimated regression coefficients from linear regression models testing for association between pack-years/cigarettes per day and polymorphic deletion CNVs on chr 3p26 in COPDGene & ARIC African Americans

	Phenotype	Estimated β	Std.Errors	p-value
COPDGene AA subjects	Pack-years	0.097169	0.025847	0.0002 ***
	Cigarettes/day	0.425393	0.116155	0.0003 ***
ARIC AA subjects	Pack-years	0.054717	0.034674	0.1149
	Cigarettes/day	0.177984	0.145275	0.2208

Regression model for COPDGene included gender, age and global admixture scores as covariates; regression model for ARIC study included gender, age and first 6 principal components as covariates.
